# Nucleus tractus solitarii is required for the development and maintenance of phrenic and sympathetic long-term facilitation after acute intermittent hypoxia

**DOI:** 10.3389/fphys.2023.1120341

**Published:** 2023-02-09

**Authors:** Daniela Ostrowski, Cheryl M. Heesch, David D. Kline, Eileen M. Hasser

**Affiliations:** ^1^ Department of Biomedical Sciences, University of Missouri, Columbia, MO, United States; ^2^ Dalton Cardiovascular Research Center, University of Missouri, Columbia, MO, United States; ^3^ Department of Biology, Truman State University, Kirksville, MO, United States; ^4^ Department of Medical Pharmacology and Physiology, University of Missouri, Columbia, MO, United States

**Keywords:** splanchnic sympathetic nerve activity, phrenic nerve activity, sympathorespiratory coupling, peripheral chemoreflex, muscimol

## Abstract

Exposure to acute intermittent hypoxia (AIH) induces prolonged increases (long term facilitation, LTF) in phrenic and sympathetic nerve activity (PhrNA, SNA) under basal conditions, and enhanced respiratory and sympathetic responses to hypoxia. The mechanisms and neurocircuitry involved are not fully defined. We tested the hypothesis that the nucleus tractus solitarii (nTS) is vital to augmentation of hypoxic responses and the initiation and maintenance of elevated phrenic (p) and splanchnic sympathetic (s) LTF following AIH. nTS neuronal activity was inhibited by nanoinjection of the GABA_A_ receptor agonist muscimol before AIH exposure or after development of AIH-induced LTF. AIH but not sustained hypoxia induced pLTF and sLTF with maintained respiratory modulation of SSNA. nTS muscimol before AIH increased baseline SSNA with minor effects on PhrNA. nTS inhibition also markedly blunted hypoxic PhrNA and SSNA responses, and prevented altered sympathorespiratory coupling during hypoxia. Inhibiting nTS neuronal activity before AIH exposure also prevented the development of pLTF during AIH and the elevated SSNA after muscimol did not increase further during or following AIH exposure. Furthermore, nTS neuronal inhibition after the development of AIH-induced LTF substantially reversed but did not eliminate the facilitation of PhrNA. Together these findings demonstrate that mechanisms within the nTS are critical for initiation of pLTF during AIH. Moreover, ongoing nTS neuronal activity is required for full expression of sustained elevations in PhrNA following exposure to AIH although other regions likely also are important. Together, the data indicate that AIH-induced alterations within the nTS contribute to both the development and maintenance of pLTF.

## Introduction

Activation of the peripheral chemoreflex by decreased arterial oxygen increases ventilation and sympathetic nervous system activity (Marshall, 1994; Guyenet, 2000), responses critical to the maintenance of oxygen delivery and homeostasis. Exposure to repetitive bouts of hypoxia induces multiple forms of plasticity within the cardiorespiratory system, including prolonged increases in respiratory motor output and sympathetic nerve activity (long term facilitation, LTF) during normoxia. In addition, individuals exposed to intermittent hypoxia undergo gradually greater respiratory and sympathetic responses (progressive augmentation) to arterial chemoreflex activation both during and after the AIH exposure (Fuller et al., 2000; Dick et al., 2007; Xing and Pilowsky, 2010; Mateika and Sandhu, 2011).

Rodents subjected to acute intermittent hypoxia (AIH) express respiratory LTF primarily *via* increased amplitude of phrenic nerve activity (i.e., phrenic LTF, pLTF) (Mitchell et al., 2001; Mitchell and Johnson, 2003). AIH also induces sympathetic LTF (sLTF), characterized by a sustained increase in sympathetic nerve activity (SNA) often without significant changes in arterial pressure (Dick et al., 2007; Xing and Pilowsky, 2010). These responses are associated with plastic alterations within neuronal networks controlling the cardiorespiratory system that are critical for adaptations to both acute and chronic physiologic stimuli. Although there is evidence for the involvement of multiple regions within the CNS in pLTF and sLTF (Mitchell et al., 2001; Xing et al., 2014; Shell et al., 2016), the specific pathways and mechanisms involved in LTF or enhancement of chemoreflex responses remain unclear. Furthermore, the underlying mechanisms that initiate or maintain pLTF, sLTF or progressive augmentation may differ.

Afferent input and its central processing likely play a role in pLTF and sLTF. Intermittent hypoxia, especially when chronic, induces LTF in chemoafferents (Peng et al., 2001; Prabhakar and Kumar, 2010). Also, repeated intermittent carotid sinus nerve stimulation produces pLTF in the absence of hypoxia (Hayashi et al., 1993) and carotid body denervation attenuates both pLTF and sLTF (Bavis and Mitchell, 2003; Kim et al., 2016). Nevertheless substantial LTF remains even after carotid body denervation (Bavis and Mitchell, 2003), indicating a role for additional mechanisms. The nucleus tractus solitarii (nTS) in the dorsomedial medulla of the brainstem is the first termination site of chemoafferent fibers (Jordan and Spyer, 1986; Finley and Katz, 1992). Neurons within the nTS integrate, modulate and transmit afferent information to other CNS sites involved in cardiorespiratory control (Andresen and Kunze, 1994; Dampney, 1994; King et al., 2012). The nTS expresses a wide array of neurotransmitters, neuromodulators and receptor types and undergoes plasticity in response to these neuromodulators and changes in afferent input, including chemoafferent input due to hypoxia (Bonham et al., 2006; Kline et al., 2007; Kline, 2010; Almado et al., 2012; Ostrowski et al., 2014; Mifflin et al., 2015; Shell et al., 2016). Neurons in the nTS alter their firing in response to high frequency carotid sinus nerve stimulation (Mifflin, 1997) and during LTF (Morris et al., 2001) and also display intrinsic hypoxic sensitivity (Powell et al., 2009). Furthermore, optogenetic stimulation of nTS neurons induces pLTF and sLTF (Yamamoto et al., 2015), implying that direct nTS activation is capable of generating LTF, although whether this plays a role in LTF induced by AIH is not known. The nTS has reciprocal projections to multiple cardiorespiratory regions in the CNS involved in LTF. Thus, activation of the nTS and plastic changes within the nTS in response to AIH may influence cardiorespiratory activity *via* alterations in neuronal function in a variety of brain regions through multiple neural circuits.

Given its pivotal role in processing chemoafferent input, we hypothesized that exposure to AIH induces pLTF and sLTF at least in part *via* mechanisms within the nTS. Furthermore, because the nTS exhibits prolonged plasticity in response to changes in afferent input (Kline et al., 2007; Chen et al., 2009a; Kline, 2010), we also hypothesized that the nTS is important in the maintenance of sustained pLTF and sLTF. Data indicate that inhibition of the nTS with the GABA_A_ receptor agonist muscimol prevented the development of LTF due to AIH and reversed established LTF. Together these data demonstrate that nTS activity is necessary for both the initiation and maintenance of LTF.

## Materials and methods

Experiments were conducted on 41 adult male Sprague-Dawley rats (Envigo, Indianapolis, IN), weighing 284 ± 7 g. Animals were housed in temperature (21–24°C) and light (12:12 h light-dark cycle)-controlled facilities and provided food and water *ad libitum*. Studies were performed in accordance with American Physiological Society guidelines and the NIH “Guide for the Care and Use of Laboratory Animals” and approved by the Animal Care and Use Committee of the University of Missouri.

### Surgical procedures

Rats were anesthetized using Isoflurane (5%, induction; 2–3% maintenance, in 100% O_2_; Aerane, Baxter, Deerfield, IL). Femoral venous and arterial catheters (PE-10 fused to PE-50, A-M systems) were inserted to enable drug administration and measurement of arterial pressure, respectively. Mean arterial pressure (MAP) and heart rate (HR) were determined using a PowerLab data acquisition system (Version 8, ADInstruments, Colorado Springs, CO). The trachea was cannulated, and rats were mechanically ventilated (60–65 breaths per min; 683 Harvard apparatus) with O_2_-enriched room air. Arterial blood gases were measured (Osmetech OPTI CCA) and ventilation adjusted for each animal to maintain blood gases above the apneic threshold as previously described (Ling et al., 2001). Arterial hemoglobin oxygen saturation was monitored continuously (MouseOx, Starr Life Sciences, Oakmont, PA). Rectal temperature was monitored and maintained at ∼38**°**C (Tele-Thermometer, Simpson Electric, Lac du Flambeau, WI). Bilateral cervical vagotomy was performed to prevent entrainment of phrenic motor output with the ventilator. The left splanchnic nerve and the right phrenic (Phr) nerve were isolated *via* a retroperitoneal or ventral cervical approach, respectively, placed on bipolar silver wire electrodes (.005’’ Bare, .0070’’ coated, A-M Systems), and covered in silicone elastomer (Kwik-Cast, WPI, Sarasota, FL) (Foley et al., 1999; Ostrowski et al., 2014; Ruyle et al., 2019). The recorded phrenic nerve was crushed distally and the contralateral phrenic nerve was cut. Nerve activity was amplified (1000x), filtered (30–3000 Hz, P511, Grass Technologies, West Warwick, RI), rectified and integrated using a root mean square converter (time constant: phrenic = 100 ms; splanchnic = 28 ms). The integrated sympathetic nerve activity signal was electronically averaged. Background electrical noise was defined as the signal between bursts of activity or following euthanasia (Matott et al., 2017). The recorded nerve activity minus noise was defined as splanchnic sympathetic nerve activity (SSNA) or phrenic nerve activity (PhrNA). The basal values of SSNA, PhrNA amplitude, and minute PhrNA prior to the initiation of the experiment were defined as 100%.

Rats were placed in a stereotaxic apparatus (Kopf Instruments, Tujunga, CA) and the brainstem exposed *via* a partial occipital craniotomy as previously described (Mueller and Hasser, 2006; Ostrowski et al., 2014; Ruyle et al., 2019). Following completion of surgery, Isoflurane was gradually withdrawn and rats were progressively converted to Inactin anesthesia (100 mg/kg i.v., 20 mg/kg i.v. supplements as required). Animals were paralyzed using gallamine (8.3 mg/kg i.v., 1–2 mg/hr i.v. maintenance). Adequate plane of anesthesia was verified regularly by lack of cardiovascular response to firm tail pinch (<5 mmHg change in MAP). Cardiorespiratory parameters were allowed to stabilize for at least 1 h prior to subsequent experimental manipulation. Arterial blood gas measurements before the experiment were not different among groups and were not significantly altered at the end of the experiments (beginning vs. end of experiment, all groups combined: PCO_2_ = 55.4 ± 2 mmHg vs. 57.1 ± 1 mmHg, *p* = 0.1351; PO_2_ = 166 ± 19 mmHg vs. 160 ± 14 mmHg, *p* = 0.3884; pH = 7.32 ± 0.01 vs. 7.29 ± 0.01, *p* = 0.1154, paired *t*-test), indicating the stability of the preparation.

### Nanoinjections

Nanoinjections (60 nL) into the nTS (level of calamus scriptorius, 0.4 mm lateral, 0.4 mm ventral) of either the GABA_A_ receptor agonist muscimol to inhibit nTS neuronal activity or artificial cerebrospinal fluid (aCSF; control) were performed as described previously (Ostrowski et al., 2014; Ruyle et al., 2019). Bilateral injections were given within 1 min of each other. In a subset of animals, the nTS was identified functionally by depressor and sympathoinhibitory responses to nanoinjection of glutamate (30 nL, 10 mM; Δ MAP = −15 mmHg ±2; n = 14; Δ SSNA = −84.9 ± 6; n = 9) prior to initiation of experiments. There was no difference in magnitude of LTF or responses to hypoxia in those animals that received glutamate injections and those that did not, and data were combined.

### Experimental protocols

#### AIH experiments

**FIGURE 1:**
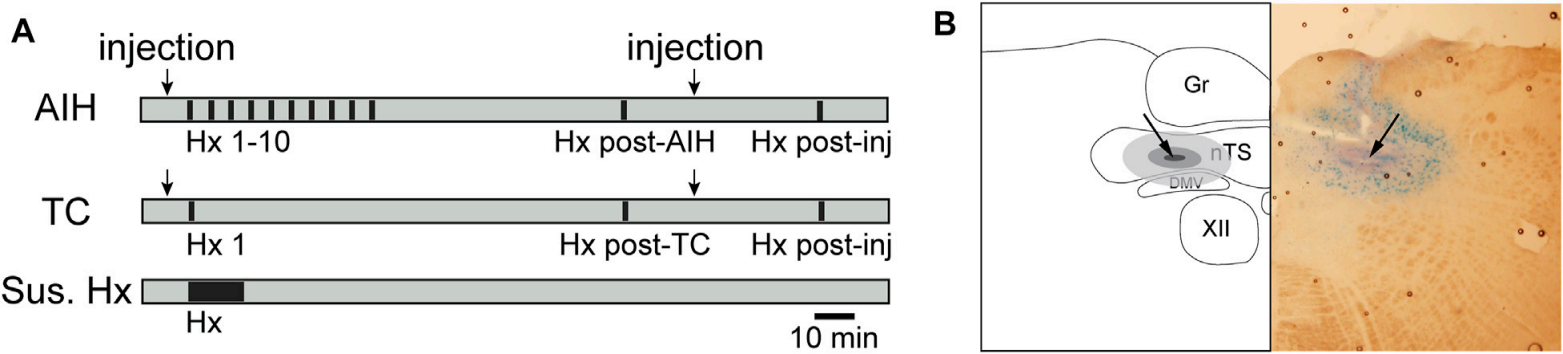
Timeline of general hypoxic experimental protocols **(A)** and example nTS injection site **(B)**. **(A)** AIH: Animals exposed to AIH undergo 10 bouts of hypoxia (Hx, 45s; black bars) separated by 5 min breathing O_2_ enriched air (grey). 1 hour following the conclusion of Hx 10, they underwent a final bout of Hx (Hx post-AIH). TC: Time control (TC) animals received only the first (Hx 1) and final (Hx post TC) bout of hypoxia. Separate groups of rats received nTS nanoinjections (arrows) of muscimol or aCSF, either prior to Hx 1 or following the AIH or TC protocol. Muscimol injections after AIH or TC were only given in rats that had no intervention or aCSF injections prior to AIH. Rats were then subjected to another bout of hypoxia (Hx post-inj). As an additional control, rats were subjected to one period of sustained hypoxia (Sus. Hx) of the same total duration as the combined time in Hx 1–10. **(B)** schematic representation (left) and photomicrograph (right) of a coronal brainstem section (−150 µm to calamus scriptorius) showing the center of the injection site (arrow). Gr, gracile nucleus; nTS, nucleus tractus solitarii; DMV, dorsal motor nucleus of the vagus; XII, hypoglossal nucleus.

Rats were exposed to acute intermittent hypoxia (AIH), consisting of 10 bouts of 10% O_2_ (45 s) every 5 min, with ventilation of O_2_-enriched room air in the intervening periods (Figure 1). This protocol is similar to others in which both pLTF and sLTF were evaluated (Blackburn et al., 2018; Xing and Pilowsky, 2010; Xing et al., 2013). Recordings continued for 1 h after the 10th hypoxic episode (Hx). A final hypoxic bout (Hx post-AIH) was administered 1 h after the last Hx of AIH. To verify the stability of the preparation and cardiorespiratory parameters, separate animals were included as time controls (TC) and were exposed to one initial hypoxic bout (Hx 1) and a final hypoxic bout approximately 2 h later (Hx post-TC) (Figure 1). An additional control group for intermittent hypoxia included rats that were exposed to the same total duration (450 s) of 10% O_2_ administered continuously (Sustained Hx, *n* = 6).

To examine the contribution of nTS activity to the development and maintenance of pLTF and sLTF, we bilaterally injected aCSF or the GABA_A_ receptor agonist muscimol (2 mM, 60 nL, Sigma-Aldrich) (Schreihofer and Sved, 1992; Mueller and Hasser, 2006) to inhibit nTS neuronal activity. Muscimol injections were performed either 5 min prior to initiating AIH (muscimol + AIH; n = 8) or following LTF development (AIH + muscimol; 1 h post-AIH; n = 9). Only rats that received either no injection or aCSF injection prior to AIH (aCSF + AIH) were given muscimol after AIH. To exclude the possibility that the nTS injections themselves had any effect on LTF, we compared PhrNA and SSNA responses to AIH between animals that received aCSF injections (60nL, pH 7.4; aCSF + AIH; n = 7) and no injections (n = 6) prior to the AIH protocol. Responses in both groups were similar, and the data were combined (*n* = 13). aCSF injections were also performed in a subset of animals following LTF development (AIH + aCSF; *n* = 5) as well as pre-TC (aCSF + TC, *n* = 4) and post-TC (TC + aCSF, *n* = 3). aCSF injections did not alter PhrNA or SSNA responses. An example of an injection site is shown in Figure 1B.

#### Baroreflex experiments

To further verify nTS inhibition following muscimol injections, in a separate set of animals (*n* = 5) we analyzed arterial baroreflex function before and after nTS injection (5 min, and 1 h and/or 2 h). To generate baroreflex curves, arterial pressure was slowly increased or decreased (1–2 mmHg/sec) by ramp i.v. infusion of the *α*_1_-adrenergic agonist phenylephrine hydrochloride (PE; 100 μg/mL) and the vasodilator sodium nitroprusside (SNP; 1 mg/mL), respectively, while monitoring MAP, HR and SSNA as previously described (Moffitt et al., 1998). After PE infusion, cardiovascular parameters were allowed to return to baseline values (within 10%) before proceeding with the SNP infusion (∼20 min).

### Data analysis

To examine the development of LTF, PhrNA [peak integrated Phr amplitude, Phr frequency and minute Phr nerve activity (Phr amplitude x frequency)] and SSNA (mean) were analyzed during periods between Hx bouts while breathing O_2_ enriched air. Time points examined included baseline (BL, prior to beginning the protocol), pre-Hx 1 (post-injection of aCSF or muscimol), immediately prior to Hx 5, 7, 10 and 15’, 30’, 45’, and 60’ post-AIH. To determine the extent to which AIH enhanced chemoreflex responses, we also examined responses to specific Hx bouts (Hx 1, 5, 7, 10, Hx post-AIH or Hx post-TC). For evaluation of LTF, PhrNA and SSNA data were normalized as a percent of the initial baseline activity (before injection/AIH; baseline = 100%). Hx responses were evaluated as the change from the baseline immediately prior to that Hx bout. In addition, changes in MAP, HR and O_2_ saturation were monitored at the above stated time points and evaluated before and after (5–10 min) nTS injections.

To evaluate sympathorespiratory coupling (Dick et al., 2004; Dick et al., 2007; Xing and Pilowsky, 2010; Ruyle et al., 2019) cycle-triggered averages of SSNA were initiated from the onset of the PhrNA inspiration (defined as 10% above the baseline value on the positive slope of integrated PhrNA). End of inspiration and beginning of expiration were determined as the peak negative slope of PhrNA. Onset of the following inspiration was defined as end of expiration. Mean SSNA was analyzed during the first and second half of inspiration (I1, I2) and expiration (E1, E2). Phr triggered averages of absolute SSNA (area under the curve *1000; Figure, 5C1) and normalized SSNA (% total activity within a cycle, where total activity = sum of I1, I2, E1, E2; Figure, 5C2) were analyzed at pre-AIH baseline (BL, 25 ± 3 Phr cycles), during Hx 1 (at peak increase of SSNA; 11 ± 2 Phr cycles), 1 h after AIH breathing O_2_ enriched air (LTF; 22 ± 3 Phr cycles) and during Hx post-AIH (12 ± 2 Phr cycles). In addition, sympathorespiratory coupling was analyzed following nTS muscimol injections (29 ± 4 Phr cycles).

Baroreflex-mediated changes in SSNA in response to increases and decreases in arterial pressure were calculated as a percentage of baseline SSNA before infusion and fit to a sigmoidal function by using a standard software package (SigmaPlot; SPSS, Chicago, IL). The following equation was used to relate SSNA to MAP: f = y0+a/(1 + exp (-(x-x0)/b)), with parameters: a—range of MAP, x0—MAP at midpoint of the curve, b—gain coefficient, y0—lower plateau of MAP changes (Kent et al., 1972; Moffitt et al., 1998).

One or Two-way ANOVA with repeated measures design was used to make statistical inferences (GraphPad Prism 9.5.0), followed by a *post hoc* test (Holm-Šídák for k = 2, Tukey for k ≥ 3) when appropriate to identify individual differences among groups and/or time points. In addition, T-tests (paired or unpaired as appropriate) were used to compare specific PhrNA and SSNA responses to hypoxia. Data that were not normally distributed (Shapiro-Wilks test, *p* ≤ 0.05, GraphPad Prism) were transformed before analysis. Differences were considered significant if *p* ≤ 0.05. All values are expressed as means ± SEM.

## Results

### AIH induces LTF of both PhrNA and SSNA and augments cardiorespiratory responses to hypoxia

The average MAP of all animals at baseline was 114 ± 2 mmHg, HR was 311 ± 6 bpm and O_2_ saturation was 99.1 ± 0.1%. Baseline hemodynamic parameters, prior to any experimental manipulations, were not different among experimental groups (Table 1). In addition, 1 h after AIH or TC neither MAP nor O_2_ saturation was altered in any group. HR remained unaltered in the aCSF + TC group, but significantly increased following AIH, sustained hypoxia or nTS muscimol treatment.

**TABLE 1:** Mean arterial pressure (MAP), heart rate (HR) and arterial oxygen saturation (O_2_ sat) at baseline (BL, left) prior to any experimental manipulations and 1 h following AIH or TC (right) of experimental groups used in this study. There were no statistical differences among groups at BL (One-way ANOVA). Also, comparison of hemodynamic parameters over the course of the experiment (Post AIH/TC) indicate no difference in MAP or O_2_ saturation. However, HR increased in most groups following either AIH or TC. Paired *t*-test: *p* ≤ 0.05: * 1 h post-AIH or post-TC vs. BL. Animals that were analyzed for baroreflex function were not subjected to hypoxia and O_2_ saturation was not monitored.

	**MAP** [mmHg]		**HR** [bpm]		**O**_**2**_ sat [%]	
	BL	Post AIH/TC	BL	Post AIH/TC	BL	Post AIH/TC
aCSF + AIH (n = 13)	112 ± 2	110 ± 4	310 ± 11	331 ± 12*	98.9 ± 0.1	98.9 ± 0.1
aCSF + TC (n = 6)	115 ± 2	114 ± 3	298 ± 20	306 ± 1	99.2 ± 0.1	99.0 ± 0.2
muscimol + AIH (n = 6)	120 ± 6	121 ± 3	318 ± 11	354 ± 16*	98.9 ± 0.2	98.9 ± 0.3
muscimol + TC (n = 5)	108 ± 3	104 ± 4	325 ± 18	357 ± 26*	99.1 ± 0.2	98.9 ± 0.3
sustained Hx (n = 6)	112 ± 6	117 ± 3	288 ± 13	300 ± 16*	99.4 ± 0.1	99.6 ± 0.2
muscimol + baroreflex (n = 5)	115 ± 5		334 ± 9			

**FIGURE 2:**
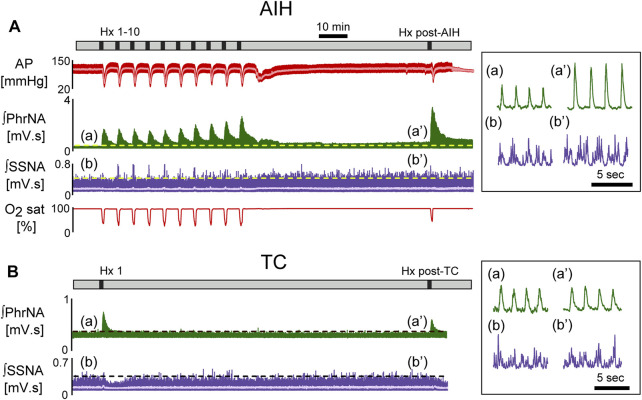
Representative recording of AP (red, MAP superimposed in light color), integrated phrenic (ʃPhrNA, green), integrated splanchnic sympathetic nerve activity (ʃSSNA, purple; mean in light purple) and O_2_ saturation (red) in response to repeated hypoxia. **(A)** AIH, Hx 1–10 and Hx post-AIH. **(B)** time control (TC). Black bars in time lines indicate periods of hypoxia. Horizontal dashed lines in traces indicate baseline PhrNA and SSNA. Insets on the right show an expanded time scale of ʃPhrNA and ʃSSNA before (a, b) and 1 h after AIH or TC (a’, b’).

Figure 2 shows a representative recording from an animal exposed to AIH [A, 10 episodes of hypoxia (45 s, 10% O_2_, separated by 5 min)] or time control (B, TC). 1 h after AIH, PhrNA, and SSNA were greater compared to pre-AIH baseline (inset), indicating pLTF and sLTF. Responses to Hx also increased during the last portion of the AIH protocol and were further enhanced during an additional hypoxic bout 1 h after AIH (Hx post-AIH). In the TC animal exposed only to an initial bout of hypoxia corresponding to Hx 1 of the AIH protocol and the final hypoxic bout (Hx post-TC) basal PhrNA, SSNA, and responses to Hx were unaltered.

**FIGURE 3:**
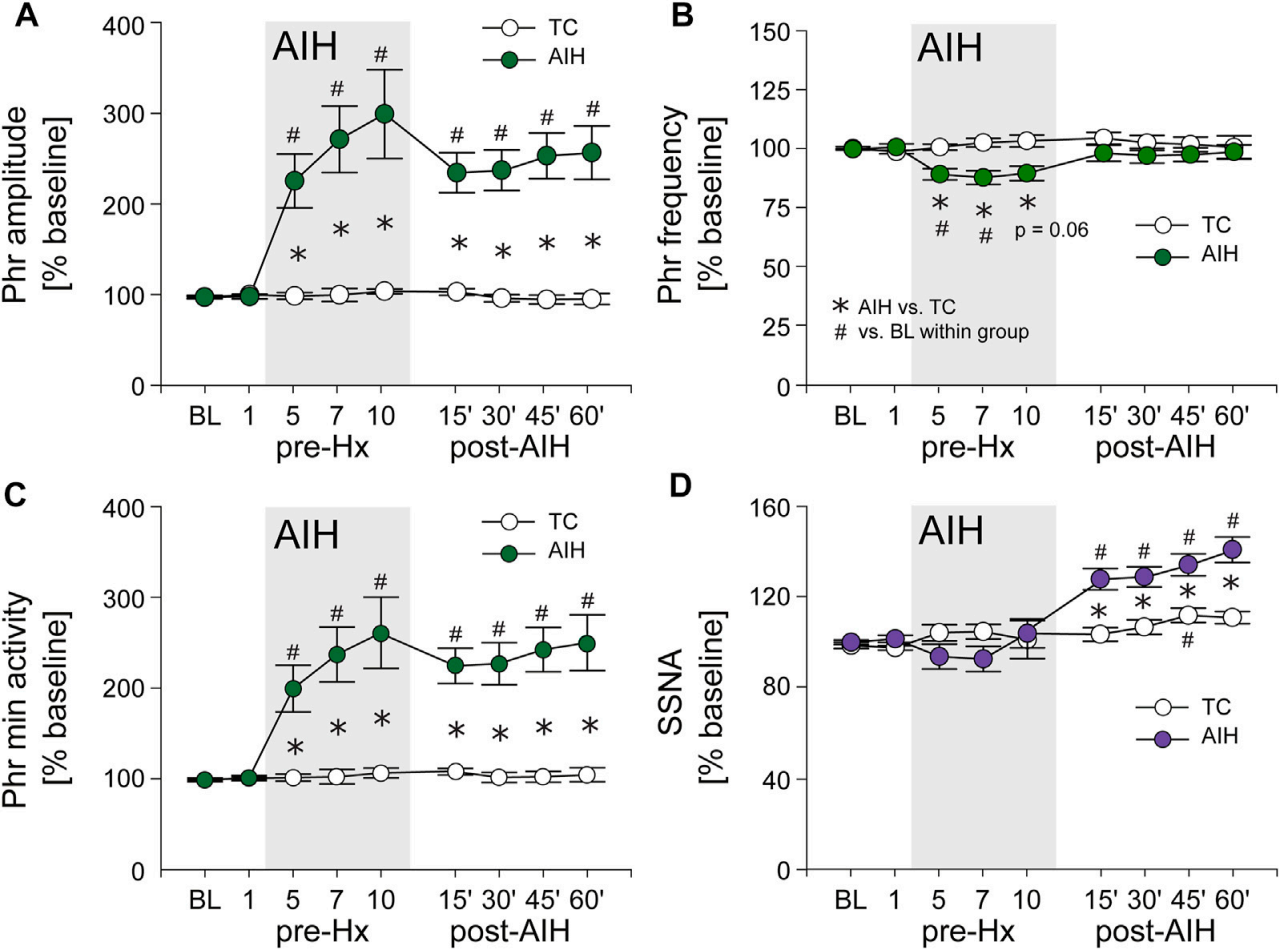
AIH induces long-term facilitation (LTF) of both PhrNA and SSNA. **(A)**, Phr amplitude; **(B)**, Phr frequency; **(C)**, Phr min activity; **(D)**, SSNA in periods of breathing O_2_ enriched air before (BL, pre-Hx 1), within (pre-Hx 5, 7, 10; grey box) and after (15′, 30′, 45′, 60′) AIH or TC protocols in animals exposed to AIH (green (PhrNA) or purple (SSNA) circles; n = 13) or TC (white circles, n = 6, PhrNA; n = 7, SSNA). Two-way repeated measures ANOVA: *p* ≤ 0.05; * AIH vs. TC, # relative to pre-AIH baseline within a group.

*Long term facilitation.* Figure 3 depicts average PhrNA and SSNA while breathing O_2_ enriched air at pre-AIH baseline (BL), during periods immediately prior to Hx 1, 5, 7, and 10, and after the AIH protocol (filled circles) or TC (white circles). In TC animals there were no significant changes in PhrNA amplitude or frequency, nor in Phr min activity while breathing O_2_ enriched air throughout the course of the experiment (Figure 3A–C) whereas SSNA increased slightly (significant at 45 min) over time (Figure 3D). In contrast, AIH produced an increase in Phr min activity during non-hypoxic periods within the AIH protocol. Elevated Phr min activity was sustained for at least 1 h following exposure to AIH (pLTF; Figure 3C), due primarily to changes in Phr amplitude (Figure 3A). Phr frequency between hypoxic bouts was lower during exposure to AIH (most likely due to posthypoxic frequency decline; see below) and returned to baseline during the post-AIH period (Figure 3B). Within the AIH protocol, SSNA during periods breathing O_2_ enriched air was not significantly altered. However, during the period following AIH exposure, SSNA increased significantly compared to the pre-AIH BL and was significantly greater than SSNA in TC animals (sLTF, Figure 3D). Thus, exposure to AIH produced LTF of both PhrNA, and SSNA, consistent with previous studies (Fuller et al., 2000; Dick et al., 2007; Xing and Pilowsky, 2010; Mitchell and Baker, 2022).

**FIGURE 4:**
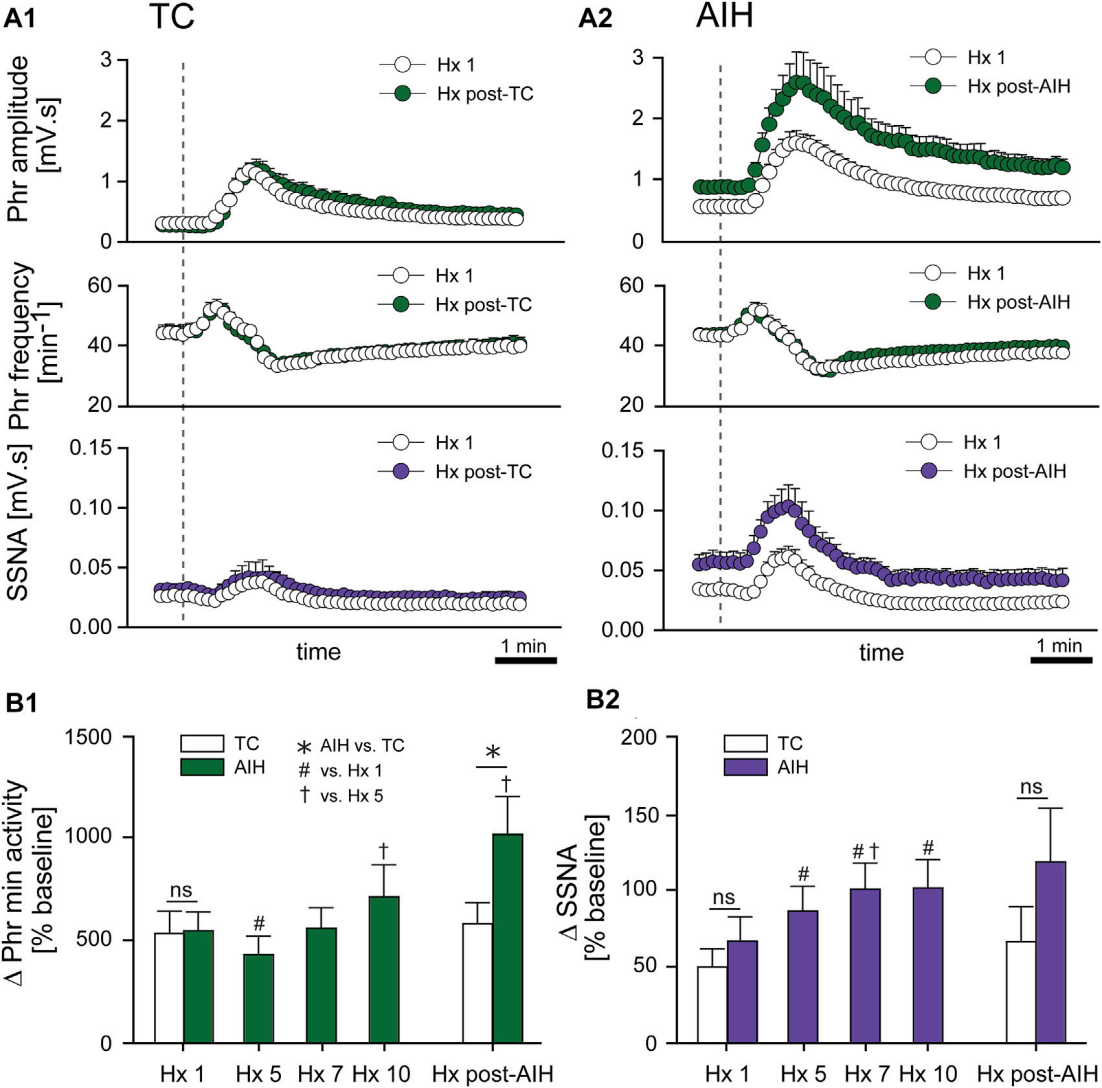
AIH increases PhrNA and SSNA responses to hypoxia. **(A)** Mean response to an initial bout of hypoxia (10% O_2_, 45 sec; Hx 1, white circles) and hypoxia following time control **(A1)**, Hx post-TC, green (PhrNA) or purple (SSNA) circles, n = 6) or after exposure to AIH **(A2)**, Hx post-AIH, green (PhrNA) or purple (SSNA) circles, n = 11). Vertical line marks the onset of the animal breathing 10% O_2_. Neither baseline PhrNA and SSNA nor the response to Hx (Hx 1 vs. Hx post-TC) was altered in TC **(A1)**. 1 h after AIH, baseline Phr amplitude and SSNA were elevated and the post-AIH Hx responses were augmented (Interaction: Phr amplitude, *p* = 0.001; SSNA, *p* = 0.003, Two-way repeated measures ANOVA) whereas Phr frequency responses were very similar **(A2)**. **(B)** Mean peak Phr min activity **(B1)** and SSNA **(B2)** in response to a given hypoxic episode. Initial Hx (Hx 1) responses were not different between groups (Phr min activity: *p* = 0.93, SSNA: *p* = 0.48, *t*-test). TC did not alter the response to hypoxia (Hx 1 vs. Hx post-TC: Phr min activity: *p* = 0.74, SSNA *p* = 0.53, paired *t*-test). The PhrNA and SSNA responses to hypoxia increased later during exposure to AIH (One-way repeated measures ANOVA: *p* ≤0.05; # relative to Hx 1, † relative to Hx 5). PhrNA during Hx post-AIH was also significantly greater compared to Hx post-TC (*p* ≤0.05, * AIH vs. TC, *t*-test).

*Progressive Augmentation.* In addition to increasing baseline activity, the change in PhrNA and SSNA during a bout of hypoxia gradually increased (progressive augmentation) during the last portion of exposure to AIH and after development of LTF (Figure 4). The time course of the PhrNA and SSNA response to the first hypoxic episode (Hx 1) and Hx post-TC or Hx post-AIH is shown in Figure 4A1, A2, respectively. In both groups, PhrNA increased during Hx, initially due to an increase in Phr frequency followed by an elevation of Phr amplitude. SSNA also increased during hypoxia. After the hypoxic episode Phr frequency initially decreased below pre-Hx levels (post-hypoxic frequency decline, (Hayashi et al., 1993)) before returning to baseline. SSNA also decreased below baseline after the end of hypoxia (Figure 4A1, A2). In time-control animals responses to a second hypoxic episode (Hx post-TC, given at the same time as Hx + AIH) were similar compared to the initial hypoxia (Figure 4A1). In contrast, exposure to AIH increased baseline PhrNA and SSNA, and significantly augmented Phr amplitude and SSNA during hypoxia post-AIH (Figure 4A2).

Mean data showing the change in PhrNA and SSNA during hypoxic bouts (Figure 4B1, B2) indicate responses to hypoxia gradually increased during the second half and after the AIH exposure. Also, the change in PhrNA due to Hx post-AIH was significantly augmented when compared directly to Hx 1 and Hx 10 (Hx 1, *p* = 0.02; Hx 10, *p* = 0.04, paired *t*-test). In contrast, in TC animals there were no significant differences in the PhrNA or SSNA response between the first and final hypoxic events. The augmented responses during and after AIH were not due to greater stimuli, since changes in O_2_ saturation were similar during Hx 1, 5, 10 and Hx post-AIH (Table 2, One-way repeated measures ANOVA). Alterations in MAP across hypoxic bouts also were similar, whereas the tachycardic response to Hx was increased following AIH.

**TABLE 2:** Mean changes in MAP, HR and O_2_ saturation during hypoxia. Responses are shown for Hx 1, 5, 10 and Hx post-AIH for AIH (left) and TC (right). Changes due to Hx were calculated relative to baseline immediately before each episode of hypoxia. AIH: One-way repeated measures ANOVA, * vs. Hx 1; TC: paired *t*-test.

	Δ MAP (mmHg)		Δ HR (bpm)		Δ O_2_ sat (%)	
	AIH	TC	AIH	TC	AIH	TC
Hx 1	−37 ± 5	−33 ± 7	+22 ± 3	+25 ± 8	−58.7 ± 1.8	−56.2 ± 9
Hx 5	−35 ± 5		+26 ± 3		−57.3 ± 2.5	
Hx 10	−33 ± 6		+31 ± 4*		−57.9 ± 3.1	
Hx post-AIH	−35 ± 6	−39 ± 5	+30 ± 5*	+18 ± 2	−51.9 ± 2.3	−55.2 ± 9

*Sustained Hypoxia.* To confirm that the effects of AIH were due to the intermittent exposure to hypoxia rather than the duration of hypoxia itself, we subjected a separate set of animals to sustained hypoxia of a similar total duration (total of 450 s, *n* = 6). PhrNA and SSNA were not significantly altered 1 h after the end of the hypoxic stimulus when compared to baseline (Phr min activity = 103 ± 6%BL; *p* = 0.25, SSNA = 116 ± 9%BL; *p* = 0.09; paired *t*-test). In addition, values were not significantly different from TC (Phr min activity: *p* = 0.98; SSNA: *p* = 0.88; One-way ANOVA) but were significantly less than after AIH (Phr min activity: *p* = 0.0004; SSNA: *p* = 0.035).

### AIH has no effect on sympathorespiratory coupling at baseline or episodes of hypoxia

**FIGURE 5:**
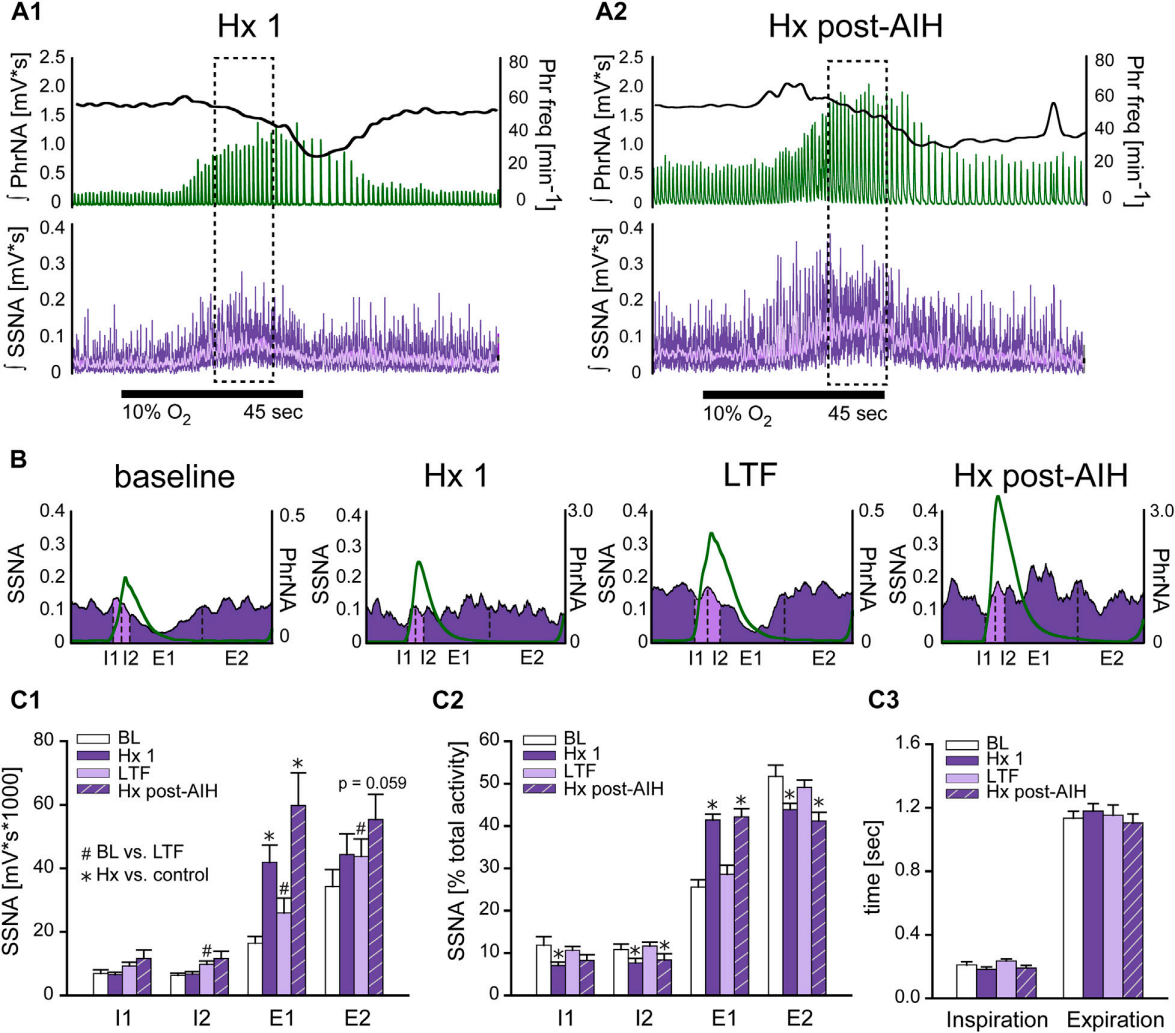
Hypoxia but not LTF alters sympathorespiratory coupling. **(A)**: Example recording of ʃPhrNA (green), Phr frequency (black, right axis) and ʃSSNA (purple, mean in light purple) during Hx 1 **(A1)** and Hx-post-AIH **(A2)**. Dashed box highlights analysis window for sympathorespiratory coupling during hypoxia, taken during the peak SSNA response; note that this occurred after the initial increase in Phr freq. Horizontal black line indicates period breathing 10% O_2_ (45 s). **(B)**: Phrenic triggered averages of SSNA from a representative animal during baseline breathing O_2_ enriched air, Hx 1, AIH-induced LTF in O_2_ enriched air (1 h after AIH) and during Hx post-AIH. Green trace is average respiratory cycle. SSNA during inspiration (I) is marked in light purple and during expiration (E) in purple. Dotted lines divide phases into first and second half of inspiration (I1, I2) and first and second half of expiration (E1, E2). **(C1)**: Mean area under the curve of phrenic triggered averages of absolute values of SSNA (n = 12). Most SSNA during pre-AIH (BL, white bars) occurred during expiration (I1 = I2 < E1 < E2; *p* ≤ 0.05, Two-way repeated measures ANOVA). During hypoxia (Hx 1, purple bars) SSNA increased in E1 and SSNA during E1 was similar to E2 (I1 = I2 < E1 = E2). 1 h after AIH in O_2_ enriched air (LTF, light purple bars) SSNA increased in almost all phases (BL vs. LTF: I1: *p* = 0.16, I2: *p* = 0.004, E1: *p* = 0.0006, E2: *p* = 0.034. One-way repeated measures ANOVA). **(C2)**: Normalized SSNA (% total activity within a given cycle). Hypoxia increased the relative amount of SSNA in E1 but decreased relative SSNA in I1, I2 and E2. No differences in the pattern of PhrNA and SSNA coupling were observed after AIH in O_2_ (BL vs. LTF: *p* = 0.89) or hypoxia (Hx 1 vs. Hx post-AIH: *p* = 0.967. **(C3)**: Mean time of I and E. Total time of inspiration and expiration was not significantly altered by LTF or during the peak SSNA response to Hx (I: *p* = 0.77, E *p* = 0.91). Two-way repeated measures ANOVA, *p* ≤ 0.05; * Hx vs. respective control (Hx 1 vs. BL; Hx post-AIH vs. LTF), # post-AIH vs. respective O_2_ level pre-AIH (LTF vs. BL; Hx-post vs. Hx 1).

To determine if changes in respiratory drive following AIH modulate sympathetic nerve activity differently, we examined the coupling between PhrNA and SSNA across the respiratory cycle while breathing O_2_ enriched air before (BL) and after AIH (LTF), as well as during hypoxia at the beginning of the AIH protocol (Hx 1), and after AIH-induced LTF (Hx post-AIH). Figure 5A represents an example recording of PhrNA amplitude and frequency, and SSNA in response to Hx 1 (A1) and Hx post-AIH (A2). Note that LTF baseline (O_2_ enriched air) PhrNA and SSNA were increased, indicating LTF, and responses during a subsequent bout of Hx were augmented. Sympathorespiratory coupling during Hx was analyzed during the peak SSNA responses (dashed box). SSNA was triggered to Phr activity and divided into 4 phases (Dick et al., 2004); the first and second half of inspiration (I1, I2) and expiration (E1, E2). Examples of Phr triggered averages of SSNA during pre-AIH baseline, Hx 1, LTF, and Hx post-AIH are shown in Figure 5B and mean data are presented in Figure 5C1, C2. To assess changes in the magnitude of SSNA during different respiratory phases, we first analyzed the overall activity (area under the curve) of phrenic triggered averages of absolute values of SSNA (Figure 5C1). As previously (Dick et al., 2004; Xing et al., 2013), at baseline (BL, white bars) most SSNA occurred during expiration (I1 = I2 < E1 < E2). During Hx 1 (Figure 5C1 purple bars) absolute values of SSNA significantly increased during E1. 1 h after AIH while breathing O_2_ enriched air, SSNA significantly increased (sLTF) compared to pre-AIH baseline in I2, E1 and E2 (Figure 5C1 light purple bars). SSNA increased further during Hx post-AIH compared to LTF, primarily during E1 (*p* <0.0001; Figure 5C1 purple striped bars).

To analyze the coupling pattern of SSNA within the different phases of I and E independent of the total amount of SSNA, we normalized SSNA to the total activity within a respiratory cycle (% total activity: absolute values of SSNA within each phase divided by the total amount of SSNA during the cycle *100). These data are shown in Figure 5C2. As expected, the pattern at pre-AIH baseline was similar to that for absolute values of SSNA, with most activity during expiration (I1 = I2 < E1 < E2; white bars). During the initial exposure to hypoxia (Hx 1, purple bars), the pattern of coupling was altered so that the % of SSNA occurring during E1 increased significantly while relative activity decreased during I1, I2, and E2. Following AIH in O_2_ enriched air (LTF, light purple bars) there was no change in SSNA pattern compared to pre-AIH BL, even though the total amount of SSNA increased. This indicates that sympathorespiratory coupling of SSNA and PhrNA was not altered by AIH. During Hx post-AIH (purple striped bars) the pattern of normalized SSNA was altered in a manner similar to that observed during Hx 1 (increase in E1). The total time of inspiration and expiration was not significantly altered at the time of peak SSNA (which occurs after the peak increase in Phr freq) during hypoxia or LTF (Figure 5C3). In summary, our results show that the pattern of sympathorespiratory coupling is shifted toward E1 by a single bout of hypoxia, and although AIH increased the absolute SSNA, sympathorespiratory coupling under baseline conditions and its alteration by hypoxia was similar following AIH.

### nTS inhibition prevents the initiation of pLTF

**FIGURE 6:**
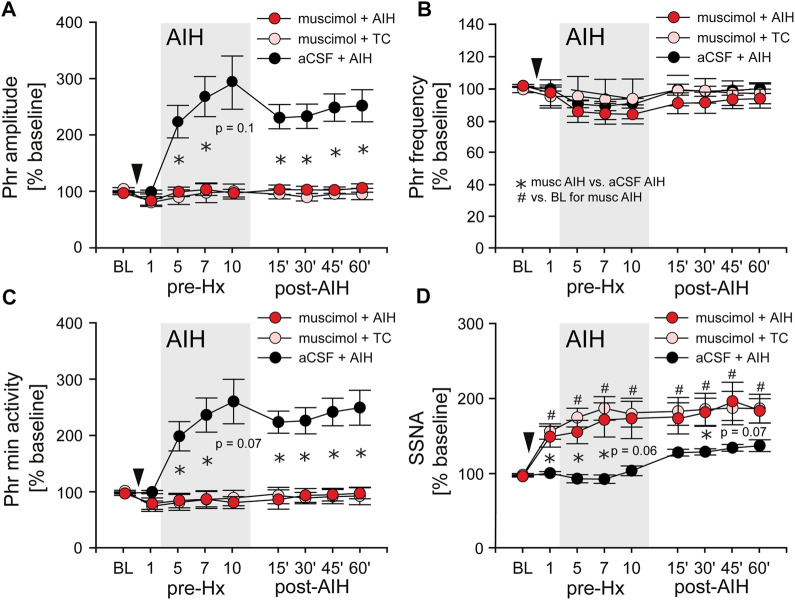
Inhibition of nTS neuronal activity prior to AIH prevents the initiation of pLTF. **(A)** Phr amplitude; **(B)** Phr frequency; **(C)** Phr min activity; **(D)** SSNA (while breathing O_2_ enriched air) before (BL, pre-Hx 1), during (pre-Hx 5, 7, 10; grey box) and after (15′, 30′, 45′, 60′) AIH in animals that received bilateral nTS muscimol injection (arrowhead) prior to either TC (muscimol + TC; pink circles, n = 5) or AIH (muscimol + AIH; red circles, n = 8; PhrNA, n = 6, SSNA). Data for animals exposed to AIH without nTS inhibition (aCSF + AIH; black circles, n = 13) prior to AIH are presented for comparison (same data as in Figure 3). Two-way repeated measures ANOVA: *p* ≤ 0.05; * aCSF + AIH vs. muscimol + AIH, # relative to pre-AIH baseline within a group.

AIH induces substantial pLTF even in carotid body denervated animals (Bavis and Mitchell, 2003) indicating the importance of other mechanisms. To test if nTS neuronal activity is required for the development of AIH-induced LTF we inhibited nTS activity by nanoinjecting aCSF or the GABA_A_ receptor agonist muscimol into the nTS 5 min prior to the initial hypoxia of AIH or TC (Figures 6A–D; arrowhead). Consistent with previous work (Schreihofer and Sved, 1992), muscimol injections produced significant increases in baseline MAP, HR and SSNA (Table 3; Figure 6D) that were similar in the AIH and TC groups, whereas aCSF injections had no effect. During the period after AIH or TC, MAP gradually decreased back to pre-injection levels while the increases in HR (data not shown) and SSNA (Figure 6D) remained elevated. In contrast, nTS muscimol injection had no significant effect on baseline PhrNA for either the TC or AIH group (Figures 6A–C).

**TABLE 3:** MAP and HR response to nTS inhibition with muscimol. Peak changes were calculated relative to baseline immediately before injection of either muscimol or aCSF. Values are means ± SEM. *p* ≤ 0.05, * compared to baseline before injection, paired *t*-test. One-way repeated measures ANOVA indicates changes due to muscimol were similar before or after AIH or TC, *p* > 0.05.

	Δ MAP (mmHg)	Δ HR (bpm)
aCSF + TC	+1 ± 1	−1 ± 2
aCSF + AIH	+2 ± 1	+2 ± 2
muscimol + TC	+30 ± 6*	+29 ± 7*
muscimol + AIH	+32 ± 3*	+21 ± 5*
TC + aCSF	+2 ± 1	+1 ± 2
AIH + aCSF	0 ± 1	−2 ± 3
TC + muscimol	+22 ± 6*	+21 ± 8*
AIH + muscimol	+23 ± 5*	+21 ± 4*

*Long term facilitation.* Inhibition of the nTS prior to AIH (muscimol + AIH) virtually abolished the increase in Phr amplitude during periods breathing O_2_ enriched air within the AIH protocol (Figure 6A; red circles). Furthermore, nTS inhibition prevented the increased pLTF during the hour following AIH. Neither Phr amplitude nor Phr min activity were different from pre-AIH baseline, or from animals with muscimol injections that were not exposed to AIH (muscimol + TC; pink circles, Two-way repeated measures ANOVA). In addition, both within and following AIH, Phr amplitude and Phr min activity were significantly less in muscimol rats than in animals with aCSF injections prior to AIH (aCSF + AIH; black circles; same data as in Figure 3). nTS inhibition had no effect on Phr frequency at baseline or due to AIH, and Phr frequency was not significantly different from that in the aCSF + AIH group (Figure 6B). The elevated SSNA after nTS muscimol injections was sustained, but was not increased further by AIH (Figure 6D). However, the level of SSNA following nTS muscimol was greater than AIH-induced sLTF and not different between muscimol + AIH and muscimol + TC (*p* = 0.19, Two-way repeated measures ANOVA).

**FIGURE 7:**
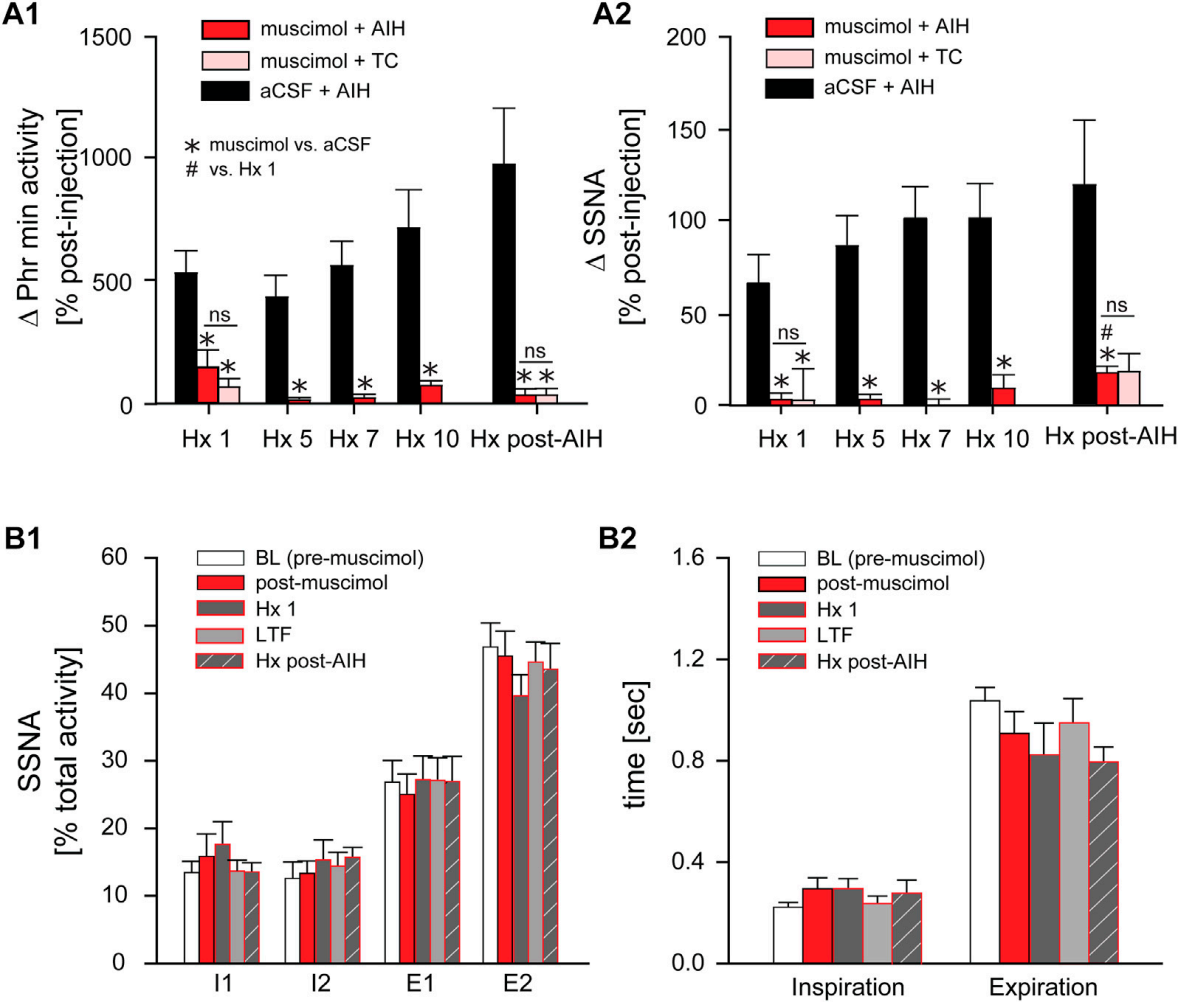
Inhibition of nTS activity prior to AIH inhibits PhrNA and SSNA responses to Hx with no changes in sympathorespiratory coupling. **(A)**: Change in PhrNA **(A1)** and SSNA **(A2)** during Hx 1, 5, 7, 10 and Hx post-AIH. Animals received bilateral nTS muscimol injections prior to either AIH (red bars, n = 8; PhrNA, n = 6, SSNA) or TC (pink bars, n = 5; PhrNA, n = 3, SSNA). Changes due to hypoxia were calculated relative to the appropriate pre-Hx baseline. Data for animals exposed to AIH (black bars) without nTS inhibition prior to AIH are presented for comparison (same data as in Figure 4B). Muscimol injections blunted PhrNA and SSNA responses to Hx. No differences were observed between muscimol + AIH and muscimol + TC at Hx 1 or Hx post-AIH (Phr min activity: Hx 1: *p* = 0.38, Hx post-AIH: *p* = 0.99; SSNA: Hx 1: *p* = 0.89, Hx post-AIH: *p* = 0.65, *t*-test). Two-way ANOVA for repeated measures: *p* ≤ 0.05; * aCSF + AIH vs. muscimol + AIH or muscimol + TC, # relative to Hx 1. **(B)**: Phrenic triggered averages of normalized SSNA (% total activity within a given cycle, **(B1**) and mean time of inspiration and expiration **(B2)** at baseline (BL, prior to muscimol injection; white bars), post-muscimol injection (red bars), during Hx 1 (grey bars), 1 h after AIH (LTF, light grey bars) and Hx post-AIH (grey striped bars). Most SSNA during baseline occurred during expiration (I1 = I2 < E1 < E2; *p* ≤ 0.05, One-way ANOVA). Muscimol injections did not change SSNA pattern in O_2_ enriched air either prior to (BL) or after AIH (LTF). However, nTS inhibition prevented changes in pattern during Hx 1 compared to periods of O_2_ enriched air, and Hx post-AIH (*p* > 0.1 for all). Inspiration and expiration time was not different following muscimol (Insp: *p* = 0.20; Exp: *p* = 0.23). One-way repeated measures ANOVA.

**FIGURE 8:**
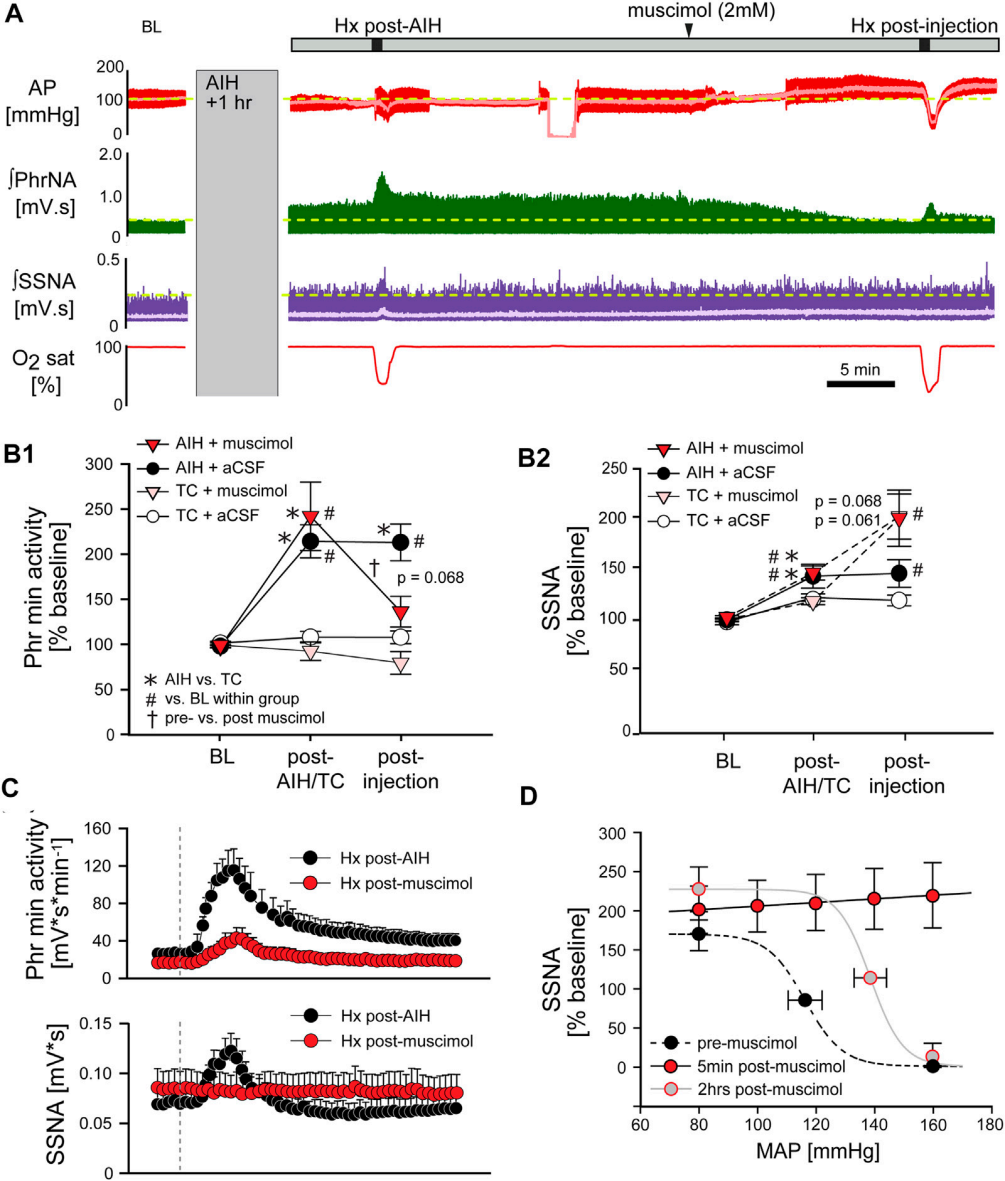
Inhibition of nTS activity following AIH reverses pLTF. **(A)** Example recording of AP (red, MAP superimposed in light red), ʃPhrNA (green) and ʃSSNA (purple, mean in light purple) at baseline (BL), LTF (1 h after AIH), during Hx post-AIH, in response to bilateral nTS injection of muscimol after LTF development and during Hx post-injection of muscimol. **(B)** Phr min activity **(B1)** or SSNA **(B2)** during periods in enriched O_2_ in animals injected with muscimol or aCSF after either AIH or TC. Following AIH, Phr min activity and SSNA were significantly elevated. Subsequent nTS inhibition with muscimol (AIH + muscimol; red triangles, n = 9) reduced Phr min activity whereas control injections of aCSF (AIH + aCSF; black circles, n = 5) had no significant effect. In TC rats, injections of either muscimol (TC + muscimol; pink triangles, n = 5) or aCSF (TC + aCSF; white circles, n = 3) did not alter Phr min activity. nTS muscimol significantly increased SSNA to similar levels following either AIH or TC. Two-way repeated measures ANOVA, *p* ≤ 0.05; * AIH vs. TC, # relative to BL within a group, † post-AIH vs. post-injection for AIH-muscimol group. **(C)** Mean responses to hypoxia post-AIH before (black circles) and following injection of muscimol post-AIH (red circles, n = 9). Dashed vertical line marks the onset of the animal breathing 10% O_2_. After injection of muscimol chemoreflex function was significantly inhibited (Interaction: Phr min activity, *p* < 0.001; SSNA, *p* < 0.001, Two-way repeated measures ANOVA). **(D)** Baroreflex function prior to muscimol injections (black symbols), 5 min after muscimol injections (red symbols, n = 5) and after a recovery period of 2 hrs (grey symbols, n = 3).

*Progressive Augmentation.* Figure 7A shows the change in Phr min activity (A1) and SSNA (A2) in response to Hx 1, 5, 7, 10, and Hx post-AIH of animals that received nTS muscimol prior to AIH (muscimol + AIH; red bars) or prior to TC (muscimol + TC; pink bars). Changes in Phr min activity or SSNA were calculated relative to baseline immediately before the given hypoxic bout. For comparison we included the responses of Phr min activity and SSNA of animals that underwent AIH in the absence of muscimol injections (black bars; same data as in Figure 4B). Although blockade of nTS activity had no effect on Phr min activity under basal conditions (Figure 6C) muscimol significantly attenuated the Phr min activity response to Hx (similar to data shown in Figure 8C), consistent with inhibition of the peripheral chemoreflex (Figure 7A1). Furthermore, after nTS inhibition, the response to Hx did not increase during the period of AIH, and PhrNA Hx post-AIH was similar to Hx 1 (Figure 7A1). In addition, there were no differences in the PhrNA response between AIH and TC muscimol groups at Hx 1 or Hx post-AIH, and both were significantly less than aCSF + AIH. Similar effects were observed for the SSNA response to Hx following muscimol. While muscimol increased baseline SSNA (Figure 6D), nTS inhibition effectively abolished SSNA responses to Hx and responses were similar throughout AIH (Figure 7A2). After nTS muscimol the SSNA response to Hx post AIH was greater than Hx 1 but remained significantly reduced compared to AIH + aCSF. Also, the SSNA responses to Hx were similar in the muscimol + AIH and muscimol + TC groups. After muscimol, MAP decreased to a greater extent during Hx (MAP during Hx 1 decreased to: aCSF, 77 mmHg ±4; after muscimol, 51 mmHg ±4; *p* = 0.0003, *t*-test), likely due to inhibition of the arterial baroreflex.

*Sympathorespiratory Coupling.* We also analyzed coupling between SSNA and PhrNA following nTS inhibition Figures 7B1, B2). At baseline (prior to muscimol injection) the SSNA pattern was similar to that described above (see Figure 5), with equal activity during the phases of inspiration and most activity during expiration (I1 = I2 < E1 < E2; Figure 7B1, white bars). Muscimol increased SSNA (Figure 6D) but had no effect on the pattern of sympathorespiratory coupling during normoxia as a percentage of total activity (Figure 7B1, red bars). nTS muscimol markedly tempered the SSNA response to hypoxia (Figure 7A2). In addition, inhibition of nTS activity also prevented Hx-induced changes in SSNA pattern both before (Hx 1) and after AIH (Hx post-AIH; Figure 7B1, dark grey and striped dark grey bars). Total time of inspiration and expiration was not significantly altered by nTS muscimol injection during hypoxia (Hx 1 and Hx post-AIH) or LTF (Figure 7B2).

### nTS inhibition reverses pLTF

*Long term facilitation.* The above data suggest that processing within the nTS is required for the development of pLTF during AIH. To determine if the nTS also is important for the maintenance of LTF we inhibited nTS neuronal activity by nanoinjecting muscimol after AIH, following the development of LTF. Figure 8A shows an example recording of MAP, PhrNA, SSNA, and O_2_ saturation from an animal that was exposed to AIH with subsequent bilateral nTS muscimol injection after LTF had developed (arrowhead). As under baseline conditions prior to AIH, nTS muscimol increased MAP, HR and SSNA (Table 3; Figure 8A). In contrast to its minimal effects at baseline, however, inhibition of the nTS during LTF decreased the elevated PhrNA toward the pre-AIH level primarily due to decreased Phr amplitude. Mean data for Phr min activity during periods in O_2_ enriched air for both the AIH- and TC-groups are shown in Figure 8B. As above, AIH induced a sustained increase in Phr min activity (pLTF) that was not altered by subsequent nTS nanoinjection of aCSF (AIH + aCSF, black circles, Figure 8B1). In contrast, inhibition of nTS activity after AIH significantly decreased Phr min activity (AIH + muscimol; red triangles) and min activity also was reduced compared to the AIH + aCSF group (*p* = 0.068, Two-Way repeated measure ANOVA). The decrease in Phr min activity was sustained for the remainder of the monitoring period (20–40 min). Nevertheless, Phr min activity remained elevated when directly compared to its baseline (*p* = 0.056, *t*-test) and its TC (*p* = 0.04, *t*-Test). Like its effects under baseline conditions, after TC muscimol had no significant effect on Phr min activity (Figure 8B1; TC + muscimol; pink triangles).

AIH increased SSNA (sLTF, as in Figure 3D) similarly in both the AIH + muscimol or AIH + aCSF groups (Figure 8B2). Subsequent inhibition of the nTS produced a further increase in SSNA whereas SSNA was not affected by aCSF injection. nTS inhibition after TC increased SSNA to a level similar to inhibition after AIH, while aCSF again had no effect (Figure 8B2). Muscimol injections after TC also increased MAP and HR (Table 3). As with its effects on Phr min activity and SSNA, aCSF following either AIH or TC had no effect on hemodynamic variables.

*Hypoxic response.* We also evaluated the effect of nTS inhibition post-AIH on cardiorespiratory changes during an additional bout of hypoxia. Mean data indicate that, like its effects prior to AIH, nTS inhibition significantly blunted but did not abolish the increase in Phr min activity in response to Hx (Figure 8C, top) due primarily to blunted Phr amplitude (see Figure 8A). The SSNA response to hypoxia (Figure 8C, bottom) also was markedly diminished by nTS inhibition following muscimol injections.

*Arterial Baroreflex.* The increased MAP, HR and SSNA following nTS muscimol injections were likely due to inhibition of the arterial baroreflex pathway. To further verify inhibition of the nTS, in a separate set of animals arterial baroreflex control of SSNA was completely blocked (5 min; red circles) after injection of muscimol into the nTS (Figure 8D). Two hours after nTS muscimol (grey circles), the baroreflex curve tended to be (but was not significantly) shifted to higher pressures (midpoint; n = 3, *p* = 0.106, paired *t*-test) compared to the curve before muscimol (black circles).

## Discussion

Exposure to AIH produces sustained elevations in phrenic and sympathetic nerve activity (pLTF and sLTF) during normoxia and progressive increases in hypoxic reflex responses (progressive augmentation) during or after AIH (Mifflin et al., 2015; Fuller et al., 2000). These processes indicate neuroplasticity but the mechanisms and neurocircuitry involved, including the role of the nTS, are not fully understood. Also, pLTF, sLTF, and progressive augmentation may occur independently of each other and their mechanisms may differ (Xing and Pilowsky, 2010; Xing et al., 2014). The nTS is critical to basal and reflex cardiorespiratory regulation, and it exhibits considerable plasticity that is essential to homeostasis and adaptations or maladaptations to multiple physiological and pathophysiological stresses. This study tested the hypothesis that the nTS is vital to the progressive augmentation of chemoreflex responses and the initiation and maintenance of elevated phrenic and sympathetic nerve activity following AIH. Our data confirm and extend previous findings that AIH exposure induces both pLTF and sLTF, and progressively augments phrenic and sympathetic nervous system responses to successive episodes of hypoxia. SSNA remained entrained to respiration, and the pattern of sympathorespiratory coupling while breathing O_2_ enriched air or during hypoxia was not altered by AIH. We now report that inhibition of nTS neuronal activity prior to AIH exposure prevented the development of pLTF. SSNA was increased by nTS inhibition to a greater extent than AIH-induced sLTF and there was no further elevation during or following AIH exposure. Furthermore, following the development of LTF due to AIH, nTS inhibition substantially reduced the magnitude but did not eliminate pLTF. Together these findings demonstrate that mechanisms within the nTS are critical for the initiation of pLTF during AIH. Moreover, ongoing nTS neuronal activity is required for full expression of sustained elevations in PhrNA following exposure to AIH and this activity may be inhibited by GABA. Together, the data indicate that AIH-induced alterations within the nTS contribute to both the development and maintenance of pLTF. Thus, it is likely that the degree of excitation or inhibition of nTS neurons affects the magnitude of LTF in response to AIH.

### nTS inhibition and cardiorespiratory function

Inhibition of nTS neuronal activity involved in cardiorespiratory control was accomplished by nanoinjection of the GABA_A_ receptor agonist muscimol at the level of calamus scriptorius. Muscimol injection markedly increased MAP and SSNA, consistent with the observed blockade of arterial baroreflex control of SNA (Andresen and Kunze, 1994; Guyenet, 2006). Sympathetic nerve activity exhibits strong respiratory modulation, although the pattern of sympathorespiratory coupling varies with the specific nerves recorded, the type of preparation and species (Dick et al., 2004; Zoccal et al., 2008; Xing and Pilowsky, 2010; Molkov et al., 2014). In the current study the majority of SSNA during normoxia occurred during expiration. Inhibiting nTS neurons markedly increased the overall magnitude of SSNA but did not alter its pattern of respiratory modulation during normoxia. These data support previous work indicating that basal respiratory entrainment of sympathetic activity is not altered by peripheral chemoreceptor denervation (Zoccal et al., 2008), and are consistent with the concept that this modulation is related to interactions among sympathetic and respiratory neurons in the ventrolateral medulla, possibly *via* inputs from the pons (Haselton and Guyenet, 1989; Mandel and Schreihofer, 2006; Baekey et al., 2008; Molkov et al., 2014). Thus, nTS neuronal activity suppresses basal sympathetic nervous system activity but is not required for entrainment of SSNA to respiration under baseline conditions.

In contrast to SSNA, nTS inhibition produced only minor effects on baseline PhrNA. Nevertheless, blocking nTS neuronal activity significantly blunted peripheral chemoreflex mediated increases in PhrNA and essentially eliminated the SSNA elevation due to hypoxia. Compared to baseline, when the nTS was intact the hypoxia-induced increase in SSNA occurred primarily in early expiration, as previously described (Dick et al., 2007; Xing and Pilowsky, 2010; Xing et al., 2013). Along with reducing the tonic increase in SSNA during hypoxia, nTS neuronal inhibition also eliminated the hypoxia-induced change in pattern of sympathorespiratory coupling. In addition, the decrease in arterial pressure during hypoxia was enhanced by muscimol, likely due to blockade of baroreflex compensation. Together, these data support previous studies (Andresen and Kunze, 1994; Chitravanshi et al., 1994; Tabata et al., 2001; Moreira et al., 2006; Favero et al., 2011) indicating that nTS neurons are critical to the maintenance of baseline arterial pressure but overall have only small effects on basal respiration. Current data also indicate that nTS neuronal activity is not required for baseline sympathorespiratory coupling, consistent with the lack of a change in pattern due to carotid chemoreceptor denervation (Zoccal et al., 2008). However, nTS neuronal activity is critical to both arterial baroreflex and peripheral chemoreflex function (Andresen and Kunze, 1994; Colombari et al., 1996; Braga et al., 2007; Guyenet, 2014) and is necessary for changes in sympathorespiratory coupling due to chemoreceptor activation by hypoxia.

### nTS inhibition prevents the development of LTF due to AIH

Data from the current study indicate that exposure to AIH produces both pLTF and sLTF, and SSNA continues to be entrained to respiration after AIH, supporting previous work (Bach and Mitchell, 1996; Cutler et al., 2004; Wadhwa et al., 2007; Dick et al., 2007; Xing and Pilowsky, 2010; Puri et al., 2021; Mitchell and Baker, 2022). Multiple anesthetic regimens have been used when examining these phenomena (Dick et al., 2007; Dale-Nagle et al., 2010; Xing and Pilowsky, 2010; Blackburn et al., 2018). Current data demonstrate that LTF also can be elicited under Inactin anesthesia and using gallamine as a paralytic, affirming the robustness of the effect. Development of pLTF is dependent upon the intermittent nature of the stimulus (Baker and Mitchell, 2000; Baker-Herman and Mitchell, 2002) and due primarily to an effect on phrenic amplitude (Bach and Mitchell, 1996; Dick et al., 2007; Xing and Pilowsky, 2010; Yamamoto et al., 2015). Furthermore, our data now demonstrate that development of pLTF is dependent on nTS neuronal activity, because nTS inhibition eliminated the initiation of phrenic LTF during the AIH exposure. Following nTS muscimol injection, Phr amplitude and Phr min activity while breathing O_2_ enriched air during and after the AIH period were unaltered from both the pre-AIH baseline and from time control rats. Muscimol also eliminated the decrease in Phr frequency during the AIH exposure, likely due to a lack of post-hypoxic frequency decline, associated with the reduced PhrNA response to Hx. Direct activation of nTS neurons *via* local intermittent optogenetic stimulation produces both pLTF and sLTF (Yamamoto et al., 2015), consistent with the idea that the nTS may contribute to the development of LTF. However, it was not determined if nTS neurons directly contribute to AIH-induced LTF and other nuclei beyond the nTS could have played a role. The current studies demonstrate that nTS neuronal activity is necessary for the initial development of pLTF during exposure to AIH.

The nTS is the first termination site of carotid body chemoafferents (Jordan and Spyer, 1986; Finley and Katz, 1992) and is critical to the processing of chemoreceptor information (Mifflin, 1992; Kline, 2010). Carotid sinus nerve stimulation in the absence of hypoxia induces respiratory LTF (Millhorn et al., 1980; Fregosi and Mitchell, 1994) and AIH-induced LTF is blunted after carotid sinus denervation (Bavis and Mitchell, 2003; Sibigtroth and Mitchell, 2011; Xing et al., 2013; Xing et al., 2014). Thus, nTS neuronal inhibition may eliminate the development of pLTF in part by blocking nTS processing of chemoafferent activity. However, a substantial AIH-induced pLTF response persists even after chemoreceptor input is removed and it has been proposed that central factors play a role, possibly associated with increased spinal cord hypoxia due to the decrease in arterial pressure during hypoxia (Bavis and Mitchell, 2003; Sibigtroth and Mitchell, 2011; Xing et al., 2014; Perim et al., 2020). Nevertheless, factors within the nTS likely contribute to the remaining pLTF observed after chemoafferent denervation, because current data show that nTS inhibition effectively abolishes pLTF altogether despite the greater hypotensive response to hypoxia. One possibility is a direct effect of hypoxia on nTS neurons, as oxygen-sensing neurons are present in several brain regions, including the nTS (Powell et al., 2009). However, the nTS is not a simple relay of afferent input, but rather is critical in integration and modulation of that information, and exhibits several forms of plasticity (Bonham et al., 2006; Mifflin, 1992; Andresen and Kunze, 1994; Kline, 2008; Kline, 2010). Thus, it is likely that the nTS undergoes plastic changes in synaptic processing due to hypoxia or altered chemoafferent activity during AIH and these changes within the nTS then contribute to the development of pLTF directly or by influencing other cardiorespiratory brain regions.

In addition to preventing the development of pLTF our nTS muscimol injections markedly attenuated the increase in PhrNA due to hypoxia, although a small response persisted. Nevertheless, after nTS inhibition AIH did not induce progressive augmentation of the remaining PhrNA response to hypoxia; the increase in PhrNA to subsequent bouts of hypoxia during or after AIH exposure was not greater than the response to the initial hypoxic episode. Progressive augmentation as well as the cellular mechanisms of LTF vary depending upon the AIH protocol including the magnitude of the stimulus (Dale-Nagle et al., 2010; Nichols et al., 2012; Devinney et al., 2013; Puri et al., 2021; Marciante et al., 2022; McGuire et al., 2022). For example, if the initial response to hypoxia is too great (Fregosi and Mitchell, 1994) or if the intensity of the hypoxic stimulus is too small (Nichols et al., 2012), progressive augmentation may not be observed. Thus, it is possible that the small PhrNA response to hypoxia after nTS inhibition may not have been sufficient to induce further augmentation of PhrNA responses. Regardless, our data suggest that nTS neuronal function is required for progressive augmentation of the hypoxic ventilatory response.

Inhibition of nTS neuronal activity produced a large, sustained increase in SSNA, likely due at least in part to blockade of arterial baroreflex function and the prolonged effects of muscimol (Gao et al., 2019). Exposure to AIH had no additional effect on the level of SSNA; elevated SSNA after nTS muscimol was similar in animals subsequently exposed to either TC or AIH. However, the magnitude of SSNA after muscimol was greater than that due to AIH in the absence of nTS muscimol. Thus, it is difficult to determine the effects of nTS on initiation of sLTF with certainty, although the similar SSNA after muscimol in AIH and TC rats is consistent with the concept that AIH-induced sLTF was eliminated by nTS inhibition.

### nTS inhibition partially reverses pLTF

Although the data suggest that nTS neuronal activity is required for its development, it is possible that the mechanisms sustaining pLTF during normoxia after AIH may differ. Carotid body chemoreceptors exhibit sensory long-term facilitation following intermittent hypoxia, in particular CIH (Peng et al., 2003; Cummings and Wilson, 2005; Prabhakar and Kumar, 2010). Thus increased chemoafferent input could play a role. However, carotid body denervation does not eliminate pLTF (Bavis and Mitchell, 2003; Sibigtroth and Mitchell, 2011), indicating the important contribution of other mechanisms. The nTS is a likely candidate due to its substantial plasticity in response to changes in afferent signaling, including with intermittent hypoxia (Kline, 2008; Almado et al., 2012; Costa-Silva et al., 2012) and because acute intermittent stimulation of nTS neurons can induce LTF in the absence of hypoxia (Yamamoto et al., 2015). In the current study nTS inhibition after TC did not significantly alter PhrNA, similar to its effects prior to initiation of AIH and further confirming minimal effects of the nTS on basal PhrNA. Importantly however, inhibition of nTS neuronal activity following the development of AIH-induced pLTF strikingly reduced the magnitude of elevated Phr min activity during periods of normoxia. These data strongly support the concept that nTS activity, at least in part, is necessary to maintain increased PhrNA following AIH. Taken together, it is likely that plasticity of nTS processing provides a sustained drive that is required for the prolonged elevation in PhrNA after AIH exposure.

Despite the effect of nTS inhibition to markedly reduce pLTF after AIH, PhrNA remained increased above baseline and TC. These data indicate that mechanisms other than those within the nTS also contribute to the maintenance of pLTF. The nTS projects to multiple brain sites involved in cardiorespiratory control, and thus plasticity within many regions of the forebrain, hindbrain or spinal cord may play a role, and may in fact also be necessary for maintenance of LTF (Andresen and Kunze, 1994; Kc and Dick, 2010; Xing et al., 2014; Shell et al., 2016). In addition, mechanisms may differ for pLTF and sLTF. For example, well-studied processes within the phrenic motor nucleus are key to the development and maintenance of pLTF (Baker-Herman and Mitchell, 2002; Devinney et al., 2015), and the cellular mechanisms vary depending on the characteristics of the AIH protocol (Dale-Nagle et al., 2010; Nichols and Mitchell, 2021). The PVN contributes to elevated SNA after chronic intermittent hypoxia (CIH) (Kc and Dick, 2010; Sharpe et al., 2013) and to sLTF but not pLTF following AIH (Blackburn et al., 2018). The importance of the PVN involves critical connections with the median preoptic nucleus, subfornical organ and organum vasculorum lamina terminalis (Cunningham et al., 2012; Shell et al., 2016; Kim et al., 2018) which likely mediate the role of angiotensin II in LTF (Mifflin et al., 2015; Kim et al., 2018). The region of the rostral ventrolateral medulla (RVLM) also appears to be important in intermittent hypoxia-induced cardiorespiratory changes (Kc and Dick, 2010; Knight et al., 2011; Silva and Schreihofer, 2011; Moraes et al., 2013), possibly related to inputs from orexin neurons since orexin neuron-ablated mice exhibit attenuated LTF (Kim et al., 2018). Regardless of the neurocircuitry involved and the importance of other brain regions, it appears that plasticity within the nTS provides a substantial sustained contribution to maintenance of the AIH-induced pLTF, as nTS inhibition prevents the development of LTF and the majority of the elevated PhrNA after AIH is reduced by nTS inhibition. It is likely that at least some of that response is mediated by its projections to other brain regions that could then play a direct role or perform a permissive function even when the nTS is subsequently inhibited.

The specific mechanisms within the nTS that influence the initiation and maintenance of AIH-induced LTF were not examined in the current study. Multiple neurotransmitters and neuromodulators as well as glia and nTS neuronal properties influence nTS function and may contribute to its plasticity, contributing to changes in autonomic and cardiorespiratory control (Bonham et al., 2006). For example, GABA plays a critical role in nTS processing *via* both pre-and postsynaptic mechanisms, and changes in nTS GABA signaling may influence altered cardiorespiratory function associated with exercise as well as cardiovascular deconditioning and hypertension (Landulpho et al., 2003; Zhang and Mifflin, 2010; Lima-Silveira et al., 2020; Martinez et al., 2022). Altered GABA signaling could be direct, or may be mediated by modulating the effects of other transmitters such as glutamate or substance P transmission (Abdala et al., 2003; Chen et al., 2009b; Zhang and Mifflin, 2010; Abdala et al., 2012). Similarly, altered signaling of many neurotransmitters/neuromodulators including glutamate, glycine, catecholamines, nitric oxide and many others could play a role. Furthermore, it is possible that altered nTS processing in AIH may involve similar processes of plasticity as occur in CIH (Kline, 2008; Kline, 2010; Mifflin et al., 2015; Shell et al., 2016). Exposure to CIH has multiple effects on nTS neurotransmission and neuronal function. CIH increases excitation of nTS neurons and their overall response to afferent input (Kline et al., 2007; Kline, 2010). CIH also induces alterations in nTS glutamate receptor function (de Paula et al., 2007), reduced K_ATP_ channel expression and currents (Zhang et al., 2008) and a reduced number of active synapses (Almado et al., 2012). In addition, nTS excitatory amino acid transporter (EAAT) membrane expression and function is reduced after CIH (Martinez et al., 2020). Together these changes produce increased nTS excitability. Alterations in nTS function are initiated relatively early in the progression to CIH and if similar plasticity occurs during AIH, they could contribute to the development and/or maintenance of LTF, and to progressive augmentation.

In summary, the current study supports and expands on previous work indicating that exposure to AIH results in both phrenic and sympathetic LTF, and induces progressive augmentation of responses to successive episodes of hypoxia. We show that neuronal activity within the nTS is required for the development of phrenic LTF due to AIH. In addition, the progressive augmentation of PhrNA responses to hypoxia during AIH was eliminated due to nTS neuronal inhibition. Also, nTS activity is not necessary for the basal pattern of sympathorespiratory coupling under baseline conditions but is required for altering the pattern of coupling during hypoxia. Finally, ongoing activity of nTS neurons is critical for the maintenance of elevated PhrNA after the development of AIH-induced LTF, although other sites within the CNS also likely play a role. Future studies are required to determine the synaptic and neural mechanisms by which the nTS influences these plastic changes in cardiorespiratory function. Changes associated with chronic exposure to intermittent hypoxia eventually can lead to sympathoexcitation and hypertension (Prabhakar and Kumar, 2010; Shell et al., 2016). Alternatively, repeated exposure to AIH may have therapeutic effects, including the ability to reduce hypertension in individuals with obstructive sleep apnea (Panza et al., 2022), or improve motor function in both respiratory and non-respiratory muscles in people with neuromuscular disorders (Vose et al., 2022). It is critical to understand and differentiate the mechanisms, including the role of the nTS, contributing to the detrimental effects of intermittent hypoxia and those that mediate the protective, potentially therapeutic effects of exposure to AIH.

## Data Availability

The raw data supporting the conclusion of this article will be made available by the authors, without undue reservation.
